# Characterization and stability evaluation of nanoencapsulated epoxylignans

**DOI:** 10.12688/f1000research.13047.2

**Published:** 2018-07-30

**Authors:** Yusnita Rifai, Radhia Riski, Gemini Alam, Magdalena Litaay, Latifah Rahman

**Affiliations:** 1 Pharmaceutical Chemistry Laboratory, Faculty of Pharmacy, Hasanuddin University, Makassar, 90245, Indonesia; 2 Biology Department, Faculty of Mathematics and Natural Sciences, Hasanuddin University, Makassar, 90245, Indonesia

**Keywords:** nanocapsules, Eudragit RL 100, Glioma, Piper nigrum

## Abstract

3',6-dimethoxy-3'',4''-(methylenedioxy)-2,5-epoxylignan-4'-ol (DMEO), an epoxylignan isolated from *Piper nigrum*, has currently captured attention for its potential antitumor effect. However, low stability is limiting its therapeutic application. The application of nanocapsulation would be the main strategy for overcoming this problem. DMEO-loaded nanocapsules were prepared by an emulsion-diffusion method using Eudragit RL 100 (at concentrations of 1, 1.5 and 2%) and polyvinyl alcohol. As the polymer content increased, the encapsulation efficiency and mean particle size also increased. After 6 months of storage at 25°C (0% RH), no crystalline peaks were observed in the diffraction patterns of all nanocapsules, thereby suggested that the physical stability of nanoencapsulated DMEO was not affected by the concentration ratio of the polymer−stabilizer combinations.

## Introduction

The hedgehog (Hh) pathway is required for the growth and proliferation of various cancers ^1^. The signaling begins with the binding of Hh protein ligand to its membrane receptor Ptch, which represses the activity of Smoothened (Smo). Smo in turn promotes the expression of the GLI (Glioma-associated oncogene) family of transcription factors, leading to tumor development ^2^. The direct association of GLI with a specific binding site (5’-GACCACCCA-3’) in the promoter region of the target gene has been reported to contribute to pancreatic and prostate cancer cells ^3^. We previously reported Hedgehog/GLI inhibitors from various plants ^4– 6^. We also isolated five lignan *s ((*8 R*,8’R*)-9-hydroxy-3,4-dimethoxy-3’,4’-methylenedioxy-9,9’-epoxylignan, kusunokinin, haplomyrfolol, dihydroclusin) including a new epoxylignan dmeo from *Piper nigrum* using the immobilization of GLI-GST on carboxylic acid magnetic dynabeads. DMEO was confirmed to have Hh signaling inhibitory activity and to be selectively cytotoxic against PANC1. Meanwhile the synthetic epoxylignan of DMEO related compound was reported to inhibit the mRNA expression of protein patched homolog (Ptch) in human pancreatic cancer cells (PANC1) and thus is considered to be a prospective drug candidate to treat cancer related to the GLI signaling pathway. However, poor solubility remains the main limitation of DMEO ^7^, hence making drug administration *in vivo* difficult. The nanoencapsulation of DMEO is one of the ways of overcoming the problem.

Nanoencapsulation techniques are particularly important to protect drugs from degradation in biological fluids and improve their penetration into cells. The techniques are also beneficial for hydrophobic molecules because the ultra-dispersed pharmaceutical dosage forms that nanoencapsulation provides allow rapid drug dissolution ^8^. The nanoencapsulation of DMEO within a suitable polymer is considered to be a good way to ameliorate its poor solubility, because the polymer acts as a rate-controlling membrane to obtain the desired controlled release. The physical stability of pure compounds remains the greatest challenge for pharmaceutical scientists seeking to exploit higher solubility properties. A gold standard revealed by the International Council for Harmonization (ICH) stated that physical stability tests of compounds should be performed within accelerated (6 months) and/or long-term (12 months) storage conditions ^9^. The physical stability was examined using powder X-ray diffraction (PXRD) analysis.

The objective of this study was to characterize the physical stability of DMEO-loaded nanocapsules, which were optimized by varying the polymer concentration to obtain stable spherical particles. The most stable particles would result from the best concentration ratio between polymers and stabilizers, ultimately improving the dissolution rate of poorly water soluble drugs.

## Methods

### Materials

Eudragrit RL 100 and polyvinyl alcohol were purchased from Sigma-Aldrich Ltd. (St Louis, MO, USA). DMEO was obtained from the Pharmaceutical Chemistry Laboratory of Hasanuddin University (Indonesia). Methanol, ethyl acetate, acetonitrile, chlorophorm and demineralized water were purchased from Merck, Indonesia. All chemicals and solvents were of analytical or pharmaceutical grade.

### Preparation of nanocapsules

Nanocapsules were prepared by an emulsion-diffusion method using Eudragit RL (ERL, Merck Ltd) at various concentrations (1%, 1.5% and 2%). Briefly, the ERL polymer (100, 150 and 200 mg) were dissolved respectively in 10 mL of ethyl acetate saturated with water. Each of this organic phase was then emulsified with 40 mL of aqueous phase, saturated with ethyl acetate, containing 300 mg of Polyvinyl alcohol (PVA) using a high speed homogenizer (ultra-turax T 25, Germany) at 1500 rpm for 60 minutes. Deionized water (150 mL) was then added to the emulsion to induce the diffusion of ethyl acetate into the continuous phase leading to the formation of nanocapsules. The organic solvent and the water phase were evaporated under reduced pressure to obtain a concentrated suspension of 40 mL.

### Preparation of DMEO-loaded nanocapsules

DMEO was previously synthesized and characterized as a white powder ^10^. 10 mg of DMEO was dissolved in 10 mL of methanol, which was then added to polymeric nanocapsules. The DMEO can be encapsulated into nanocapsules at a maximum concentration of 1 mg/ml with the ratio maintained at the 1:40; 1:45; and 1:50 drug/polymer ratio. DMEO-loaded nanocapsules were dried in a desiccator until constant weight. They were then kept in a closed glass vial and stored at 25°C.

### Characterization of DMEO-loaded nanocapsules

The size of DMEO-loaded nanocapsules was analyzed by laser diffraction using a Partica LA-950 laser diffraction particle size analyzer (Horiba Ltd, Japan). Dried particles (5 mg) were dispersed in Miglylol 812 using a UP50H ultrasound processor (Hielscher, Germany) and analyzed in triplicate. The surface characteristics of the particles were observed by scanning electron microscopy (SEM) (Jeol, JSM-5600 LV, Japan) magnification at 600x. The yields of the particles were calculated by the sum of the weights of all components, discounting the content of water in the suspensions. After stirring the powders in acetonitrile for 90 minutes at room temperature followed by centrifugation and filtration (GVWP membrane, 0.45 µm, Millipore), the nanoencapsulation efficiency of DMEO was determined by UV-Vis spectroscopy (Shimadzu; 229 nm absorbance detector). The nanoencapsulation efficiency (%) of the powder was calculated from the correlation of the theoretical and experimental DMEO concentration. The onset of its crystallization was examined before and after 6 months of storage at 25°C, using an X-Ray Diffractometer (XRD-7000, SHIMADZU) with Cu K α radiation (λ = 0.154 nm). The diffraction patterns were analyzed using X’Pert Highscore Plus (version 2.2d).

## Results

**Figure 1.**
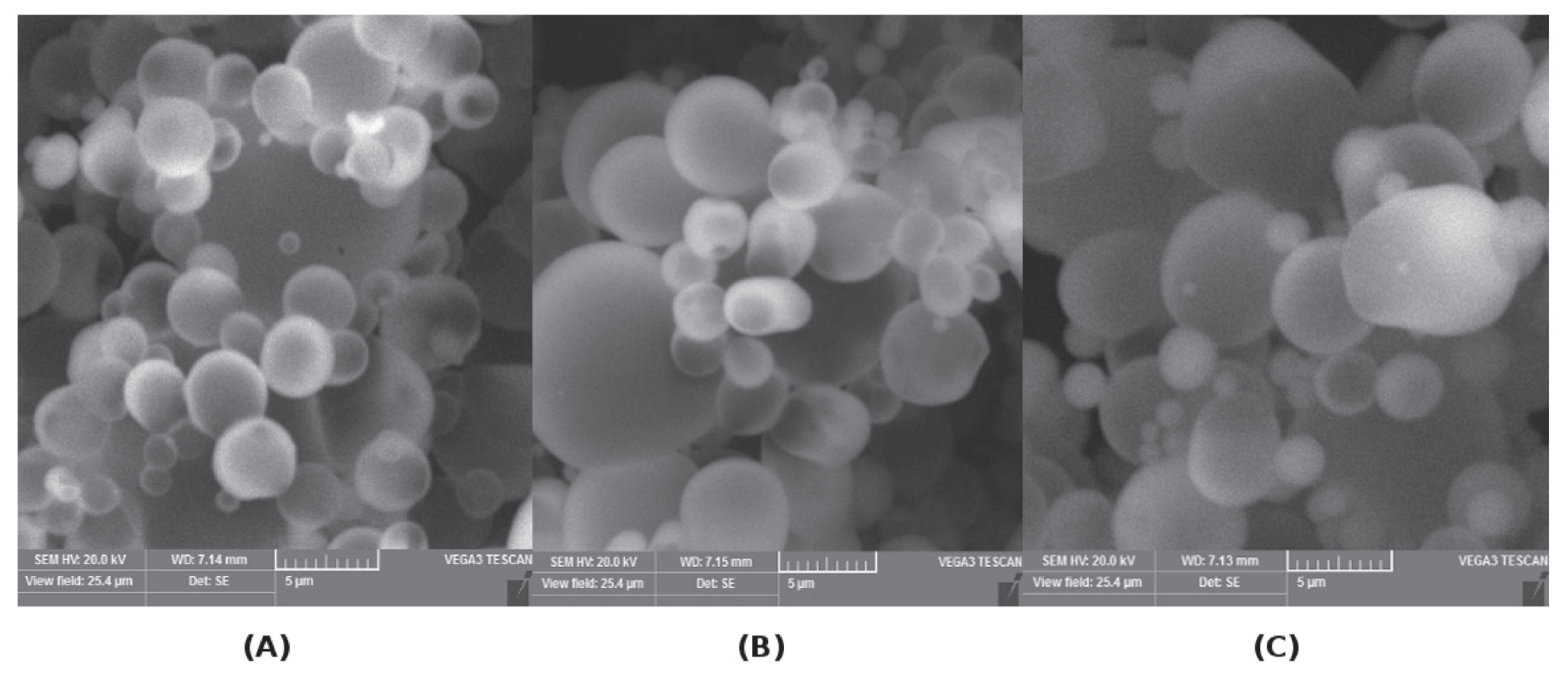
SEM images of sprayed-dried powder in formulations ( **A**) F1 ( **B**) F2 and ( **C**) F3.

SEM analysis reveals that all DMEO-loaded nanocapsules are mostly spherical ( Figure 1). As the polymer content increased, the encapsulation efficiency and mean particle size also increased, as seen in Table 1, suggesting that nanoencapsulation minimized deactivation of the drugs during the delivery process due to the protection from the polymer shell. This might ensure sufficient amounts of drug reaching the targeted areas. ERL is a copolymer of partial esters of acrylic acid containing low amounts of a quaternary ammonium group. ERL is a water-soluble polymer that plays a crucial role in controlling drug release because its water uptake characteristic contributes to the swelling of the polymers.

**Table 1.** The observed mean particle sizes and encapsulation efficiencies of DMEO-loaded nanocapsules.

Sample Code	DMEO (mg)	Polymer (mg)		Mean Particle Size (nm)	Encapsulation Efficiency (%)
		PVA	ERL		
F1	10	300	100	227.55±0.231	89.53±0.307
F2	10	300	150	253.96±0.012	89.46±0.211
F3	10	300	200	255.09±0.455	90.31±0.352

**Figure 2.**
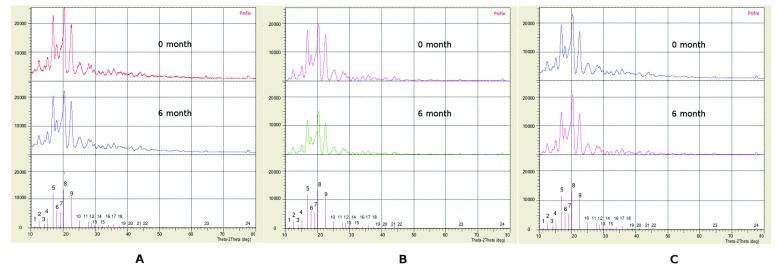
( **A**) X-ray diffraction patterns of DMEO-loaded nanocapsules of F1 after 0 and 6 months of storage ( **B**) X-ray diffraction patterns of DMEO-loaded nanocapsules of F2 after 0 and 6 months of storage ( **C**) X-ray diffraction patterns of DMEO-loaded nanocapsules of F3 after 0 and 6 months of storage.

In crystalline materials, atoms are periodically arranged, but in non-crystalline materials, atoms are randomly arranged. Figure 2 shows that the peaks of DMEO do not possess that periodicity, while the characteristic peak falls at 19.3° 2θ. After the physical mixtures were subjected to various concentrations of polymers and stabilizers, all the peaks were re-observed at 0 and 6 months of storage. No crystalline peaks were observed in the diffraction patterns, as shown in Figure 2a, 2b and 2c (upper, down), suggesting that the particles remain spherical throughout the duration of the stability study. The unchanged position of the largest peak of DMEO, which remains at 19.3° 2θ ( Figure 2a, 2b and 2c (upper, down)), indicates that there is no change in the diffraction pattern of DMEO after 6 months of storage. It is important for the DMEO particles to remain stable during 6 months of storage to ensure that the resulting DMEO-loaded nanocapsules meet the minimal standard requirements of drug formulation stability.

Raw data for particle size, particle yields, nanoencapsulation efficiencies and X-ray diffraction pattern values Click here for additional data file. Copyright: © 2018 Rifai Y et al. 2018 Data associated with the article are available under the terms of the Creative Commons Zero "No rights reserved" data waiver (CC0 1.0 Public domain dedication).

## Discussion

The polymer-based encapsulation may be beneficial regarding improved short-term physical stability. The nanoencapsulation of DMEO yielded entrapment efficiencies of 89.53±0.307, 89.46±0.211, 90.31±0.352% with particle sizes of 227.55±0.231, 253.96±0.012, and 255.09±0.455 nm, respectively. It is generally considered that higher molecular weight polymers have less entropy loss related to their fast motion, which results in a higher affinity to drug surfaces ^10^, and they therefore perhaps have better steric formation. The charge along the polymer chains can build a strong double layer surrounding the particles, granting electrostatic stabilization and therefore smaller particle size ^11^.

The results showed that the polymer concentration influenced the particle size and entrapment efficiency. The 1% polymer concentration formula (F1) fulfilled the requirement of stable nanocapsules, including spherical and uniform surface morphology. This formula exhibited the greatest percentage of nanocapsules’ weight (10.24%) but had the smallest particle size (227.55 nm). Meanwhile, F3 showed the greatest entrapment efficiency (90.31%). We chose F1, F2 and F3 for the further physical stability study using XRD to see whether the different concentrations of polymer interfered with the stability of DMEO in nanocapsules. Polymers were expected to adsorb to the surface of the nanocapsules, providing both electrostatic and steric repulsion among particles, therefore producing nanoparticles with decreased particle sizes and improved physical stability. The polymers may adsorb to the drug surfaces through several points along the polymer chains, enabling the loops and tails of the polymers to extend into the liquid medium, providing a steric effect.

The key finding in this work was that higher concentrations of polymer in the stirring solution during the production process yielded higher encapsulation efficiencies and smaller particle sizes. Tuning the polymer ratio was essential to obtaining spherical particles, without necessarily affecting the stability of the obtained nanocapsules.

## Data availability

The data referenced by this article are under copyright with the following copyright statement: Copyright: © 2018 Rifai Y et al.

Data associated with the article are available under the terms of the Creative Commons Zero "No rights reserved" data waiver (CC0 1.0 Public domain dedication). 

Dataset 1: Raw data for particle size, particle yields, nanoencapsulation efficiencies and X-ray diffraction pattern values. 10.5256/f1000research.13047.d195348 ^12^

## Supplementary material

Supplementary File 1: Structure of 3’,6-dimethoxy-3’’,4’’-(methylenedioxy)-2,5-epoxylignan-4’-ol (DMEO). ^1^H and ^13^C NMR Data of DMEO
