# Supplementary material for: Characterization and stability evaluation of nanoencapsulated epoxylignans
Source: F1000Res. 2018 Jul 30;7:253. Originally published 2018 Mar 1. [Version 2] doi: 10.12688/f1000research.13047.2 (PMC6081991; doi:10.12688/f1000research.13047.2)
Supplement: Supplementary file 2 [file f1000research-7-17172-s0001.tgz › a737e605-a0e7-4b76-ab7f-eb17db663748.doc]

**Supplemental Material**

**Characterization and Stability Evaluation of Nanoencapsulated Epoxy Lignans**

Yusnita Rifai ^1*^, Radhia Riski^1^ , Gemini Alam^1^, Magdalena Litaay^2^ and Latifah Rahman^1^

^1^	Pharmaceutical Chemistry Laboratory, Faculty of Pharmacy Hasanuddin University, Makassar 90245, Indonesia

**^2^**	Biology Departement, Faculty of Mathematics and Life Sciences Hasanuddin University, Makassar 90245, Indonesia

**Structure of 3',6-dimethoxy-3'',4''-(methylenedioxy)-2,5-epoxylignan-4'-ol (DMEO)**

 **^1^H and ^13^C NMR of DMEO**
