# Supplementary material for: Characterization and stability evaluation of nanoencapsulated epoxylignans
Source: F1000Res. 2018 Jul 30;7:253. Originally published 2018 Mar 1. [Version 2] doi: 10.12688/f1000research.13047.2 (PMC6081991; doi:10.12688/f1000research.13047.2)
Supplement: Peer review history

# Peer review: Referee response for version 2

**DOI:** 10.5256/f1000research.17172.r36598

**Published:** 2018-08-06

**Authors:** Heni Rachmawati

**Affiliations:** 1 School of Pharmacy, Research Center for Nanosciences and Nanotechnology, Bandung Institute of Technology, Bandung, Indonesia

The revised manuscript presents improved information and explanation regarding my previous comments. The manuscript is well written followed by clear figures which convince the basic findings. Although this is a small work, there are sufficient data to conclude early successful approach to improve the physicochemical lack of the active compound through encapsulation in the nanocarrier system. 

I have read this submission. I believe that I have an appropriate level of expertise to confirm that it is of an acceptable scientific standard.

# Peer review: Referee response for version 1

**DOI:** 10.5256/f1000research.14146.r33856

**Published:** 2018-05-23

**Authors:** Heni Rachmawati

**Affiliations:** 1 School of Pharmacy, Research Center for Nanosciences and Nanotechnology, Bandung Institute of Technology, Bandung, Indonesia

This article describes the strategy to improve the lack of physical characteristic of such natural active compound by developing the nanocarrier system based on polymer. The authors discussed the correlation between polymer concentration and the drug loading efficiency. The main objective as well as the concept is acceptable in term of scientific perspective. However, some lack important information in particular in the method session: how the drug-loaded nanocapsules was formed, the sample preparation on such evaluation e.g PXRD, and the detail on SEM analysis (magnification etc). The ****important data to conclude the successful approach are missing: solubility and/or dissolution rate. Also, the only PXRD data is not sufficient to conclude the stability issue of the drug-loaded nanocapsules. The description presented in the result section is not sufficient to explain the phenomenon found in this study.

I have read this submission. I believe that I have an appropriate level of expertise to confirm that it is of an acceptable scientific standard, however I have significant reservations, as outlined above.

# Response

**Published:** 2018-07-23

**Authors:** Yusnita Rifai

**Affiliations:** Hasanuddin University, Indonesia

Thanks for valuable scientific feedback. We add some more explanation in the method session of the revised manuscript associated with how the drug-loaded nanocapsules are formed, the sample preparation and the detail on SEM analysis (magnification at 600x). As for the preparation of nanocapsules, the DMEO can be encapsulated into nanocapsules at a maximum concentration of 1 mg/ml with the ratio maintained at the 1:40; 1:45; and 1:50 drug/polymer ratio.

Meanwhile, the diffraction patterns of the DMEO-loaded nanocapsules exhibit a diffraction peak at 19.3° 2θ. These results indicate that DMEO-loaded nanocapsules are not present as a crystalline state, but are probably dissolved within the nanocapsule’s core. It is noted that the only PXRD data is not sufficient to conclude the stability issue of the drug-loaded nanocapsules, the PXRD data aimed to characterize the crystallinity of DMEO in the formulated nanocapsules.

# Peer review: Referee response for version 1

**DOI:** 10.5256/f1000research.14146.r31917

**Published:** 2018-04-10

**Authors:** Rani Sauriasari

**Affiliations:** 1 Department of Pharmacy, Faculty of Pharmacy, Universitas Indonesia, Depok, Indonesia

The overall construct of the manuscript is generally concise. The authors highlight the higher concentrations of polymer in the stirring solution during the production process yielded higher encapsulation efficiencies and smaller particle sizes. However, it is better to add statistical analysis to show the correlation between concentration of polymer with encapsulation efficiency and particle size.

I have read this submission. I believe that I have an appropriate level of expertise to confirm that it is of an acceptable scientific standard.

# Response

**Published:** 2018-07-23

**Authors:** Yusnita Rifai

**Affiliations:** Hasanuddin University, Indonesia

Thanks for the valuable feedback. Statistical analysis is important to correctly interpret the data. However, because of the small number of sample formula and their repetitious treatment (n =3), we did not do any statistical analysis.
